# Recent Advances in the Pathogenesis and Management of Cast Nephropathy (Myeloma Kidney)

**DOI:** 10.1155/2011/493697

**Published:** 2011-05-04

**Authors:** Stephanie Stringer, Kolitha Basnayake, Colin Hutchison, Paul Cockwell

**Affiliations:** ^1^Renal Institute of Birmingham, University Hospital Birmingham, Birmingham B15 2TH, UK; ^2^Department of Nephrology, University Hospital Birmingham, Birmingham B15 2TH, UK; ^3^School of Immunity and Infection, University of Birmingham, Birmingham, B15 2TT, UK

## Abstract

Multiple myeloma is an incurable plasma cell malignancy that is often accompanied by renal failure; there are a number of potential causes of this, of which cast nephropathy is the most important. Renal failure is highly significant in myeloma, as patient survival can be stratified by the severity of the renal impairment. Consequently, there is an ongoing focus on the pathological basis of cast nephropathy and the optimal treatment regimens in this setting, including effective chemotherapy regimens to reduce light chain production and emerging extracorporeal techniques to remove circulating light chains. This paper bridges recent advances in the pathogenesis and management of cast nephropathy in multiple myeloma.

## 1. Introduction

The kidneys are a common target for injury in a large number of acute and chronic diseases that initially develop at nonrenal sites. Where renal injury develops as a secondary consequence of nonrenal disease, then (i) the factors that cause this injury are often complex; (ii) the involvement of the kidneys and the development of subsequent acute kidney injury (AKI) or chronic kidney disease (CKD) has a deleterious effect on subsequent clinical outcomes. The effective management of kidney disease is an important factor in improving the long-term outcomes of the affected individual. Multiple myeloma (MM) is commonly complicated by kidney injury with a major impact on long-term outcomes; therefore understanding the nature of the kidney damage and the treatment options that are available are crucial for improving outcomes for people with MM and kidney injury. In this paper we will overview the current status of basic and clinical science in this area and show how increasing knowledge in both these areas is leading to improvements in clinical outcomes.

## 2. Multiple Myeloma and the Kidneys

Multiple myeloma (MM) is a haematological malignancy characterised by clonal proliferation of plasma cells and associated with end-organ damage. There is production of a monoclonal immunoglobulin (Ig) in over 80% of those affected; each intact monoclonal Ig will include either a kappa light chain (LC) isotype or a lambda LC isotype; in the large majority of cases of intact monoclonal Ig in MM there is excess production of the relevant monoclonal LC, and in around 20% of cases there is LC monoclonality with no detectable intact immunoglobulin [1]. This detectable Ig LC is often referred to as free light chain (FLC).

The incidence of new-onset (first diagnosis) MM in the United Kingdom is around 6.5 per 100,000 in Caucasians, 10-9-18.2/100,000 in Blacks, and 3.6–6.4/100,000 in Asians [2]. At presentation at least 50% of patients will have AKI of a variable degree, and around 10% have severe AKI from which they will die within weeks unless they are treated with renal replacement therapy by dialysis [3]. The prognosis for patients with MM and AKI is worse than that for those with normal renal function [4]. This directly relates to the severity of AKI at time of presentation, such that those who require and receive dialysis treatment have a median survival of less than 12 months [5]. 

There are a number of factors that can contribute to the development of AKI in MM; these include dehydration, hypercalcaemia, and the ingestion of nephrotoxic drugs [6]. It is important to note that cast nephropathy may not be the sole pathology in patients with MM and AKI; for example, there may be concomitant amyloid or another renal pathology unrelated to MM. All these factors have the potential to trigger the development of cast nephropathy, which is the commonest cause of severe AKI. Severe AKI in MM is a major medical emergency, as it is associated with a high risk of early death. Historically, less that 20% of people with MM require dialysis at or shortly after presentation recover independent kidney function [5]. As a consequence, there is a major focus on understanding the biological basis for the development of AKI in MM. In addition, the efficacy of novel chemotherapies for promoting better outcomes is under evaluation. Finally, with the introduction of protein-permeable dialysers into clinical practice, there is continuing interest in the clinical utility of extracorporeal removal of light chains to improve renal recovery.

## 3. The Pathogenic Basis of Cast Nephropathy in Multiple Myeloma

**Figure 1:**
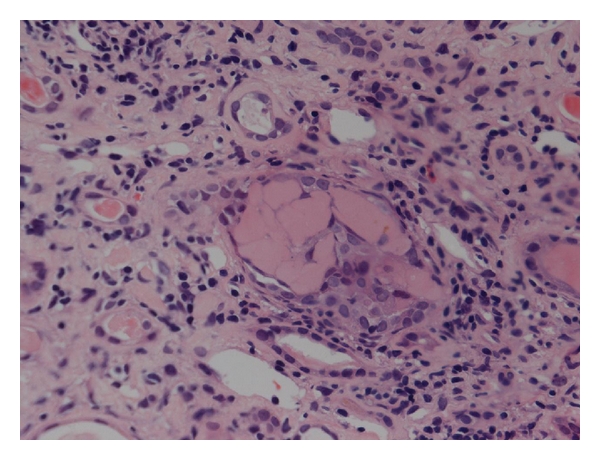
Renal biopsy showing cast nephropathy: distal tubular casts and interstitial inflammation and fibrosis.

Cast nephropathy is characterised by the presence of fractured, waxy casts formed of FLC and uromodulin (Figure 1) [7, 8]. These casts precipitate out in the distal tubules resulting in tubular obstruction; there is also associated tubulointerstitial inflammation, which may in part be related to a direct proinflammatory effect of FLC on proximal tubular epithelial cells (PTEC) [7, 8].

**Figure 2:**
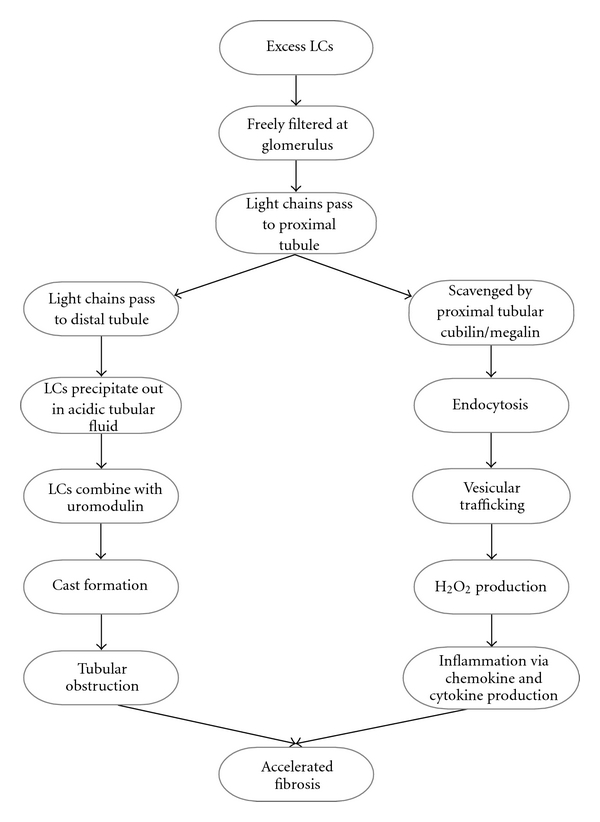
Renal handling of LCs.

 To fully understand the pathogenesis of cast formation it is necessary to understand the renal handling of FLC (Figure 2). Serum FLCs are relatively freely filtered at the glomerulus because of their size (22.5 kD for Kappa (*κ*) and 45 kD for Lambda (*λ*)) and cationic net charge; the glomerular sieving coefficient for free Kappa LC has been calculated at 0.09 [9]. As there are detectable levels of polyclonal FLC in the serum of normal individuals (*κ* at 3.3 to 19.4 mg/L; *λ* at 5.7 to 26.3 mg/L), it can be extrapolated that in health between 100–600 mg/24 hr of FLC are presented at the renal tubule; as there are minimal levels of FLC present in the urine this indicates a high capacity for reabsorption of FLC by the tubules [10]. This process of FLC reabsorption takes place at PTEC through receptor-mediated interactions, and free LCs in tubular ultrafiltrate are taken up by the tandem receptors cubilin/megalin, with subsequent endocytosis via the endosomal/lysosomal pathway; some FLC clones bind preferentially to megalin and some to cubilin [11–13]. After endocytosis the FLCs undergo vesicular trafficking, which is dependent upon acidification of the vesicle. However not all FLC digestion is confined to lysosome, and there may be some degradation of FLC bound to the PTEC membrane [12]. 

There is no evidence to date that endocytosis of polyclonal FLC activates PTEC; however there is substantial data that show a profound proinflammatory and cytotoxic potential of monoclonal FLC. There is a differential capacity of any given clone of FLC to activate PTEC to produce proinflammatory cytokines through activation of NF*κ*B; this may contribute to the inflammatory cell infiltration and accelerated fibrosis that is seen in cast nephropathy [14]. The production of these cytokines also involves mitogen-activated protein kinases (MAPKs) ERK 1/2, JNK 1/2, and p38 [11]. 

A central component of the pathogenicity of monoclonal FLC towards PTEC is mediated through the formation of hydrogen peroxide (H_2_O_2_,); in vitro this is generated after FLC endocytosis and indicates a high level of oxidative stress [15]. It has subsequently been established that H_2_O_2_ production by monoclonal FLC mediates the oxidation and activation of c-Src (a redox sensitive, nonreceptor tyrosine kinase), an obligate step in this setting for the production of MCP-1/CCL2 [16]. These and other studies show that monoclonal FLCs have a greater inflammatory effect on PTECs than other freely filtered proteins [14, 17].

The dynamics of serum FLC (sFLC) levels and renal clearance is important for the interrelationship between FLC processing by PTEC and the development of cast nephropathy. As MM evolves, the amount of clonal FLC that is present in glomerular ultrafiltrate progressively increases, ultimately to levels that overwhelm the reabsorptive capacity of PTEC. Therefore increasing amounts of FLC are present in filtrate in the loop of Henle and the distal tubule. There is then a differential capacity of any given clone of FLC to aggregate with uromodulin (Tam-Horsfall protein) to form casts [18]. One of the key determinants of cast formation is the isoelectric point (pI) of the protein. 

When the ambient pH of tubular filtrate is close to the FLC pI, the protein carries no net charge and will tend to precipitate out of solution; proteins with a pI of 5.6–7.3 tend to precipitate in the ascending limb of the loop of Henle or early distal tubule while those with a pI < 5.1 precipitate more distally (the pI of FLC has been shown to range from 3.5 to 9.9) [18, 19]. This reflects the progressive acidification of tubular fluid as it flows through the distal nephron; this consequently facilitates the precipitation of proteins with progressively lower pIs. This may have direct clinical relevance in the management of the acid-base status of the individual patient which is supported by disease model studies, where the alkalinisation of the urine ameliorated the effects of FLC on inulin clearance in rats [20].

After precipitation of FLC, cast formation occurs when uromodulin traps the FLC and a gel-like cast is formed. Uromodulin is secreted at the thick ascending limb of the loop of Henle; it is the main urinary protein in healthy individuals and has the ability to self-aggregate into a gel like substance and bind with many low molecular weight proteins [21, 22]. Uromodulin has been shown to have a specific binding site for FLC; this has been shown in animal models to be a peptide sequence [23]. When a monoclonal antibody against this peptide sequence was utilised there was a reduction in FLC binding to uromodulin, and there is great interest therefore in the potential for small molecule blockers to reduce cast formation [23, 24]. Uromodulin may also have an immunomodulatory role via the induction of inflammatory cytokines such as TNF-*α* and IL-6 from monocyte/macrophages and neutrophils [25, 26]. 

In conclusion, current concepts around the pathogenesis of AKI in MM can be summarised as follows: (i) cast nephropathy may be precipitated by dehydration, hypercalaemia, or nephrotoxic drugs; (ii) the precipitating factors for cast nephropathy may themselves cause AKI in the absence of cast formation; (iii) the direct pathogenicity of monoclonal FLC on PTEC may also precipitate AKI; this may occur in combination with cast nephropathy or (less commonly) through causing acute tubular necrosis (ATN) in the absence of cast formation. Renal impairment is more likely to be reversible with supportive measures alone when it is not related to cast formation [3].

Our understanding of the biology of clonal FLC in producing AKI in MM indicates that aggressive early reduction of FLC levels may be an important factor in promoting the recovery of kidney function. This concept is now being supported by clinical studies that clearly show a relationship between an early fall in sFLC levels and renal recovery [27, 28]. It is now clear that novel chemotherapeutic agents that are having a substantial impact on improving overall outcomes for patients with MM may be particularly important in the setting of MM and AKI, in part through early disease response.

## 4. Novel Agents and Outcomes of Patients with Renal Impairment

There are two crucial considerations in the assessment of the utility of novel chemotherapeutic agents in MM and AKI: firstly, the efficacy and safety of these agents in this setting and, secondly, the potential for these agents to reverse renal impairment. Whilst it is self-evident that an agent that rapidly reduces tumour burden should also result in an improvement in renal outcome, little work has been done to specifically address this question. This section will focus on the clinical utility in this setting of three novel agents used in both denovo and relapsed MM: Thalidomide, Bortezomib and Lenalidomide. If used in the setting of MM and AKI, these agents should probably be used in regimens that incorporated dexamethasone at cytotoxic doses; this is because they act synergistically and early work comparing vincristine and doxorubicin alone or with high-dose dexamethasone suggested that most of the plasma cell reduction was due to the high-dose dexamethasone [29, 30].  Table 1 shows those chemotherapy studies that have reported both the renal characteristics of the participants and their renal outcomes [31, 32].

### 4.1. Thalidomide

Thalidomide was first developed in the 1950s and was then used widely for numerous indications; however it was subsequently found to have devastating teratogenic effects and clinical use ceased. It was subsequently postulated that the teratogenic limb defects caused by the drug were caused by inhibition of angiogenesis, and this prompted investigations into its potential as an antitumour agent [41]. In 1965 the use of Thalidomide was first reported in a patient with MM, with a subsequent delay in disease progression. However, it was not until 1997 that patients were first recruited into a clinical trial examining the effect of Thalidomide in MM [42, 43]. Since then there have been numerous studies using the drug that have established its efficacy in MM, and it is now recommended for use in patients with MM in a range of clinical settings [44]. Thalidomide is safe in renal impairment; it is not renally excreted (the primary route of clearance appears to be hepatic), and there is no dose adjustment needed for renal impairment or in patients undergoing dialysis [45, 46]. 

Tosi et al. considered the use of Thalidomide-containing regimens on the renal outcomes of patients. They enrolled 20 patients (two who were dialysis dependent) with a serum creatinine of ≥130 *μ*mol/l and defined renal recovery as achieving a serum creatinine of <130 *μ*mol/l [38]. All patients received Thalidomide at the same dose, in combination with a range of other agents. They reported a 67% renal recovery rate, although neither of the dialysis patients recovered independent renal function [38]. More recently Thalidomide use has been reported in larger numbers of patients who have been dialysis dependent at presentation; in a study of high cut off haemodialysis 14/19 patients received Thalidomide and recovered renal function [27].

### 4.2. Lenalidomide

Lenalidomide is a derivative of Thalidomide but has a different side effect profile, it was introduced in 2004, and there has been subsequent interest in its role in the treatment of MM. There have been several large studies of Lenalidomide in relapsed or refractory MM that show excellent clinical outcomes with response rates (defined variously as complete, near complete, partial, or very good partial responses) in the region of 50–60% [47–50]. However there has been little work examining the role of Lenalidomide in patients with renal impairment, as traditionally trials excluded patients with renal impairment (to varying degrees), largely because early pharmacokinetic studies demonstrated that over 60% of the drug is excreted in the urine as a result of glomerular filtration and tubular secretion [51]. Subsequently, Chen et al. carried out a multicentre, open label study to investigate the effect of renal impairment and dialysis on the pharmacokinetics of Lenalidomide. They enrolled 30 patients, whom they stratified on the basis of renal function [52]. They confirmed that Lenalidomide is predominantly excreted by the kidneys (80% of total drug clearance) and that a creatinine clearance of <50 mls/min is the threshold at which dose modification is needed in respect of renal function [50]. For patients on dialysis there was a substantial decrease in trough concentrations postdialysis, indicating that an extradose is needed following dialysis treatment [50]. 

Dimopoulos et al. reported a subgroup analysis of the MM-009 and MM-010 trials; this analysis focused on the impact of renal impairment on safety and efficacy of Lenalidomide and also on the renal outcomes of the cohort [40]. The degree of renal impairment was quantified by the calculation of creatinine clearance by the Cockcroft-Gault formula, and patients were divided into subgroups depending upon their renal function at baseline; these were mild or no renal impairment (CrCl >60 mls/min), moderate renal impairment (CrCl 30–60 mls/min), or severe renal impairment (CrCl <30 mls/min); however all patients had to have a baseline serum creatinine of <2.5 mg/dL [39]. Renal recovery was defined as improvement in renal function as defined by transition to a subgroup with better renal function. There was no difference in disease response rates between subgroups, and response was not influenced by the degree of renal impairment. In moderate or severe renal impairment, 72% of the patients had an improvement in their renal function [40]. While individuals with very severe renal failure were excluded and definitions for renal recovery are not precise, this work suggests that Lenalidomide is safe and effective with a creatinine of up to 2.5 mg/dL and in this setting may improve renal function.

Klein et al. reported a retrospective analysis of patients with relapsed/refractory MM and renal impairment; the cohort included patients who were dialysis dependent [32]. After treatment with Lenalidomide and dexamethasone they report an improvement in renal function in 26%; patients whose renal function stabilised or improved had a higher frequency of PR than those whose renal function deteriorated [41]. 

### 4.3. Bortezomib

Bortezomib is a proteasome inhibitor that is the first in class, and it was synthesised in 1995 and was first used in humans until 1999. On the basis of a large phase II clinical trial it was recommended by the FDA for use in relapsed/refractory MM [52]. It undergoes hepatic clearance with no renal clearance, and therefore no dose adjustments are recommended for patients with impaired renal function [53]. There have now been many large studies investigating the use of Bortezomib, initially in relapsed/refractory MM and more recently in de novo MM. A number of these have analysed the drug in impaired renal function, but few consider the renal outcomes of treated patients. 

The largest study of renal outcomes is a subgroup analysis of the VISTA study; this was a randomised phase III study comparing melphalan and prednisolone (MP) to melphalan, prednisolone, and Bortezomib (VMP). However the study excluded patients with a serum creatinine >2 mg/dL (176 *μ*mol/l) so was not applicable to patients with severe AKI [31]. Despite this there was some indication of a benefit in the Bortezomib group with better renal outcomes in the patients treated with the drug (44% renal recovery in treatment arm; 34% in the control arm; *P* = .001 the time to renal recovery was also faster in the VMP arm [31].

Ludwig et al. also considered the renal outcomes of patients treated with Bortezomib; in their study it was used as part of a BDD (Bortezomib, doxorubicin, and dexamethasone) regimen; they enrolled 68 patients with light-chain-induced renal failure (defining acute renal failure as a recent decline in glomerular filtration rate (GFR) <50 mLs/min where other causes of renal failure had been clinically excluded) [37]. All patients received the same treatment regimen, though only 58 patients were available for analysis (seven died before the 2nd cycle, one discontinued after the 2nd cycle because of toxicity, one had progressive disease, and one had incomplete data) [36]. They reported complete response rates of 31% and an overall response rate of 62%, the renal outcomes of the cohort were also reported, and the median GFR increased from 20.5 mls/min (3.7–49.9 mls/min) to 48.4 mls/min (6.7–135.5 mls/min) [37]. It is important to note that the number of patients with histological evidence of cast nephropathy was small; only six patients had had a renal biopsy (of whom two were not enrolled because of a diagnosis of amyloid), and the numbers requiring dialysis at the outset and the end were also not reported.

Chanan-Khan et al. considered the use of Bortezomib in dialysis-dependent renal impairment. They retrospectively examined the records of 24 patients who were treated for MM and required dialysis at that time [33]. Of the 24 patients initially considered there was insufficient data available in six, so results on 18 patients were available for analysis [33]. Of the remaining patients, 23% were independent of dialysis at analysis (one never actually needed dialysis, two became dialysis independent after a complete response (CR) and one after a minimal response (MR)) [33]. There was no increase in adverse events reported compared to patients without renal impairment [33]. As a result the authors concluded that Bortezomib was safe and effective in patients requiring dialysis.

Encouraging evidence with Bortezomib and dexamethasone in patients with renal impairment has led to an International Myeloma Working Group recommendation that high-dose dexamethasone and Bortezomib are the recommended treatment for MM in patients with any degree of renal impairment [30]. Given the previously poor prognosis for patients with renal impairment and MM it is logical to focus on treatment with chemotherapy regimens that have a high response rate and where that response is associated with a rapid early decline in tumour (and therefore clonal FLC) load. Where Bortezomib is not available, usually as a consequence of cost, then Thalidomide should be used, again in combination with dexamethasone.

In addition to an evolution in the chemotherapy options for patients with AKI and MM, there has been a recent reevaluation of the role of extracorporeal removal of sFLC. Whilst this may seem an attractive addition to therapies that are available, it is critical to understand that this adjunctive therapy will only add benefit in the setting of effective chemotherapy. Furthermore, any proposed use must be validated by properly designed prospective randomised controlled trials.

## 5. Extracorporeal LC Removal Strategies

Plasma exchange has been variably used in clinical practice for the removal of FLC; however, there is no convincing evidence of benefit compared to treatment regimens that do not include plasma exchange. More recently, novel protein permeable dialysis membranes have been developed, and their utility in FLC removal has attracted considerable interest.

### 5.1. Plasma Exchange

Plasma exchange (PE) involves extracorporeal processing of a patient's blood to remove pathogenic substances from the plasma; dependent on the clinical indication these can include autoantibodies, cryoglobulins, or other abnormal plasma proteins or immune complexes [54]. A single plasma exchange removes approximately 75% of the patient's own plasma and the abnormal constituent in the plasma [54]. Therefore PE was considered as for the removal of remove FLCs in MM and was investigated in a series of studies. The largest and most robust study to date, reported by Clark et al., examined the outcomes of 104 patients (of whom 97 completed 6-month followup) with acute renal failure at the time of presentation of MM [55]. There were several design shortfalls in the study; these included the absence of histological diagnosis of cast nephropathy and the presence of dialysis dependency at randomisation in only 28% of those recruited [55]. The patients were randomised to receive either conventional therapy with the addition of between five and seven plasma exchanges or conventional therapy alone. The results showed that there was no benefit derived from plasma exchange by a composite end point of death, dialysis dependence, or renal function at six months [55].

Other studies of PE have shown conflicting results, but these are all limited by small sample sizes and other methodological flaws [56, 57]. Collectively, these studies raised questions about why PE is ineffective. The most important reason probably relates to the short duration of the treatment, which results in removal of FLC from the intravascular space only. As 85% of the total FLC load is in the extravascular compartment, this leads to rapid redistribution following PE of FLC into the intravascular compartment [58]. As a consequence, PE has a limited impact on the overall exposure of the kidneys to FLC. One obvious way to address is to utilise an extended treatment that removes FLC over a prolonged period of time and therefore removes FLCs that are undergoing redistribution from the extravascular to the intravascular compartment; the recent introduction of protein permeable dialysers into clinical practice has led to the practical assessment of the utility of extended extracorporeal removal of FLC in MM and AKI.

## 6. High Cut-Off (HCO) Haemodialysis

Recently, clinical researchers have utilised a newer type of larger pore dialysis membrane (the Gambro HCO 1100, initially developed for cytokine removal in critically ill patients on intensive care units by continuous dialysis modalities) and showed high clearances of both isotypes of FLC both in vitro and in vivo [59]. This finding was consistent with the physical characteristics of the dialyser, which has an effective cut-off for the removal of molecules of a molecular weight of <50 Kd. In the initial report, the efficacy of the dialyser was reinforced by significant reductions in serum FLC levels in patients with MM and renal failure on extended dialysis sessions [59]. 

This work was followed by a pilot study of HCO dialysis in MM. Nineteen patients with biopsy proven cast nephropathy and severe AKI requiring dialysis were enrolled, treated with chemotherapy regimens that included novel agents, and prescribed extended HCO dialysis sessions of up to 8 hours a day [27]. The treatment was well tolerated, and recovery of renal function to independence of dialysis was seen in 14 patients (74% renal recovery). Of particular interest in this study was the observation that there was an early and sustained reduction of sFLC levels in those patients who recovered kidney function; those patients who had early interruptions in chemotherapy did not have early reductions in FLC levels and usually did not recover independent kidney function [27]. 

These initial results have led to the development of EuLITE, a randomised controlled trial of HCO dialysis versus conventional dialysis in patients with de novo MM, biopsy proven cast nephropathy, and dialysis-dependent acute kidney injury at presentation. All patients receive chemotherapy with Bortezomib, doxorubicin, and dexamethasone. The study is ongoing and has a primary outcome measure of dialysis independence at 3 months [60].

## 7. Conclusions

Multiple myeloma remains an incurable disease although long-term outcomes are improving. However patients with AKI, particularly those requiring dialysis treatment, continue to have poor outcomes. A greater understanding of the pathogenesis of cast nephropathy may lead to successful prevention strategies and improvements in chemotherapy and the emergence of an effective method of LC removal contribute to future improvements in clinical outcomes for those affect.

## Figures and Tables

**Table 1:** Renal characteristics of chemotherapy trials where both baseline renal characteristics and outcomes are reported.

Study	Agents	Renal Inclusion criteria	Baseline renal characteristics	Renal outcomes
San Miguel et al. (VISTA) [31]	VMP versus MP	No renal exclusions	CrCl < 30 = 6% (B), 5% (Control) CrCl 30–60 = 28% (B), 50% (Control) CrCl >60 = 46 (B), 46% (control)	Reversal = GFR >60 at end from <50 at start VMP group = 44% reversal MP group = 34%

Chanan-Khan et al. [33]	B + variety of other agents	Dialysis dependence mandatory	All but 1 dialysis dependent, Median sCr = 6.8 mg/dL (3.1–12.8)? before starting dialysis No renal Bx	No clear def^n^of renal reversal 4 had “improved” renal function (1 pt never started dialysis, 2 came off dialysis after a CR and 1 off dialysis after a MR)

Kastritis et al. [34]	VAD (or similar), M + hdD or D alone Group B: hdD + T +/_ B	sCr >2 mg/dL mandatory at enrolment	sCr >4 mg/dL = 44% sCr 2–4 mg/dL = 56% 24% dialysis dependent No renal Bx	sCr <2 g/dL defined as renal reversal, 83% met this def^n^ median TTR = 1.9 mo 80% of those previously dialysed came off dialysis

Morabito et al. [35]	V + D or V + D + other agents	No renal exclusions	CrCl 51–80 = 10.3% CrCl 30–50 = 19.7% CrCl <30 = 70% 12% dialysis dependent	Reversal = normalisation of CrCl, occurred in 41% with no differences across treatment subgroups 22% of those previously dialysed came off dialysis

Ludwig et al. [36]	V, V + D or V, D + A	GFR <20 mls/min mandatory at enrolment	Mean sCr 9.05 mg/ml (5.2–12) 63% dialysis dependent No renal Bx	Median sCr fell from 9.05 to 2.1 mg/dL (0.8–2.4)

Ludwig et al. [37]	VAD	GFR <50 mls/min mandatory at enrolment	Median GFR 20.5 mls/min (3.7–49.9 mls/min) 6 renal Bx (2 showed amyloid so patients excluded)	No defined criteria for renal recovery, median GFR increased to 48.4 ml/min (6.7–135.5 mls/min), improvement in GFR correlated with tumour response

Tosi et al. [38]	T + other	sCr >130 *μ*mol/l mandatory at enrolment	Initial sCr for all listed, Median = 155 *μ*mol/l Range 131–998 *μ*mol/l	Yes, Improvement defined as sCr <130 *μ*mol/l, 12/15 responding pts had renal recovery

Dimopoulos et al. [39]	R + D	No renal exclusions	24 had baseline CrCl, 50 (13–49), 2% dialysis dependent at baseline	Recovery defined as either CrCl, 50 to >60 or movement from one subgroup to another, 42% of those with renal impairment had some improvement

Dimopoulos et al. [40]	R + D versus D	sCr <2.5 mg/dL	CrCl >60 = 71% CrCl 30–60 = 24% CrCl <30 = 5%	Improvement defined as movement from one subgroup to a better one, 72% of those who received R+D had some improvement, data for D alone not quoted

Dimopoulos et al. [40]	R + D	No renal exclusions	CrCl >80 = 56% CrCl 50–80 = 23.9% CrCl 30–50 = 12.6% CrCl <30 = 7.2% 3% dialysis dependent	26.6% had some change from one renal subgroup to another, 12.5% had deterioration of renal function

V: bortezomib, D: dexamethasone, M: Melphalan, P: prednisolone, A: doxorubicin, hD: high dose, T: Thalidomide, BMT: bone marrow transplant, R: Lenalidomide, CrCl: creatinine clearance (mls/min), Bx: biopsy, sCr: serum creatinine, CR: complete response, MR: minimal response, TTR: time to response, GFR: glomerular filtration rate.
